# How visuomotor predictability and task demands affect tactile sensitivity on a moving limb during object interaction in a virtual environment

**DOI:** 10.1098/rsos.231259

**Published:** 2023-12-13

**Authors:** Meaghan McManus, Immo Schütz, Dimitris Voudouris, Katja Fiehler

**Affiliations:** ^1^ Experimental Psychology, Justus Liebig University, Otto-Behaghel-Str. 10F, 35394, Giessen, Hessen, Germany; ^2^ Center for Mind, Brain and Behavior, University of Marburg and Justus Liebig University, Giessen, Hessen, Germany

**Keywords:** tactile suppression, somatosensory, prediction, task load, virtual reality

## Abstract

Tactile sensitivity is decreased on a moving limb compared to the same static limb. This *tactile suppression* likely reflects an interplay between sensorimotor predictions and sensory feedback. Here, we examined how visuomotor predictability influences tactile suppression. Participants were instructed to hit an approaching virtual object, with the object either never rotating, or always rotating, or rotating unpredictably, prompting related movement adjustments. We probed tactile suppression by delivering a vibrotactile stimulus of varying intensities to the moving hand briefly after the object's rotation and asked participants to indicate if they had felt a vibration. We hypothesized that Unpredictable Rotations would require upweighting of somatosensory feedback from the hand and therefore decrease suppression. Instead, we found stronger suppression with unpredictable than Predictable Rotations. This finding persisted even when visual input from the moving hand was removed and participants had to rely solely on somatosensory feedback of their hand. Importantly, we found a correlation between task demand and tactile suppression in both experiments, indicating that task load can amplify tactile suppression, possibly by downweighting task-irrelevant somatosensory feedback signals to allow for successful task performance when visuomotor task demands are high.

## 1. Introduction

When we move our arm, we do not continuously feel our loose sleeve moving against our forearm. This phenomenon of decreased tactile sensitivity on a moving compared to the same but static limb is termed tactile suppression. It has been shown in a variety of movements, from simple finger movements [1] to goal-directed actions such as bimanual reaching or grasping [2–5], and even in juggling [6], with various factors being involved (for a review, please refer to [7]).

Tactile suppression is thought to occur as the result of a feedforward mechanism that generates sensory predictions about the upcoming movement [8,9], which are compared with the actual sensory feedback associated with that movement [10]. If the prediction and feedback match, then neural activity in primary and secondary somatosensory brain regions is decreased [11,12] and tactile sensitivity on the moving limb is downregulated [8,13] so that any stimuli presented on that limb are perceived as less strong than they actually are. Accordingly, suppression is stronger if there is a predictable tactile consequence to the action compared to when there is no consequence [14]. The degree of tactile suppression has also been found to be modulated by the speed of the moving limb with stronger suppression for faster movements ([15,16]; but see also [14]), suggesting a possible effect of afferent, feedback signals on this phenomenon (but see also [17]). This is supported by the observation of tactile suppression during passive movements, when no movement plan is supposed to be formed, suggesting an additional role of peripheral mechanisms. However, the degree of tactile suppression is reduced during passive compared to active movements [18,19].

Humans use visual and somatosensory input to guide their actions to static (e.g. to pick up a glass) or dynamic targets (e.g. to pick up an item from a conveyer belt) (e.g. [20]). This requires us to determine the spatial relation between the effector and the target which needs to be continuously updated as the person or the target moves. Sudden perturbations in the object's movement are particularly challenging as they require immediate adjustments to the movement. As proposed by the optimal feedback control framework, such corrective movements have been associated with an upweighting of somatosensory feedback signals [21], which might influence associated tactile processing, as greater reliance on feedback signals can reduce tactile suppression [22,23]. In this study, we investigated tactile suppression when interacting with dynamic objects that vary in the predictability of their behaviour. We exploited the advantages of virtual reality (VR) allowing us to systematically change the object's dynamics and to assess hand motion and tactile sensitivity using the VR controllers [24]. As experiments performed in VR have been associated with higher cognitive load compared to tasks performed on a monitor or in real-world environments [25–27], probably due to increased visual complexity and unfamiliar object dynamics during interaction, we also assessed task load across our experimental conditions which varied in the dynamics of the interaction target.

The participant's task was to intercept an approaching object using a virtual sword. To alter the reliance on somatosensory feedback signals about the moving hand's position, we manipulated the predictability of the object's dynamics. Higher predictability should allow participants to plan their movement well in advance, and thereby reduce the need for online movement corrections. Meanwhile, lower predictability should result in stronger online movement corrections and thus in higher reliance on somatosensory feedback of the moving hand. If tactile suppression is modulated by the reliance on online somatosensory feedback, then we expect suppression to be lower in conditions with low compared to high predictability of the object's dynamics. Specifically, the object's orientation could change during approach which would require participants to adjust their movement trajectory to successfully hit the object. To assess movement-related tactile suppression, a vibrotactile probe stimulus of varying intensities was applied to the participant's hand shortly after the object's rotation, i.e. at the time when movement adjustments were necessary. Participants responded at the end of the trial if they felt the vibration or not.

Based on previous findings [2,5,24], we hypothesized that the probing stimuli would be perceived as weaker during movement relative to a resting Baseline condition. If the strength of tactile suppression reflects the weighing of predictive and somatosensory feedback signals [5], tactile suppression should be weaker when the object changes orientation, which may require facilitation of somatosensory feedback processing to successfully adjust the movement. This suppression should also be weaker when the object rotates in an unpredictable compared to a predictable manner [22] as movement adjustments cannot be pre-planned and need to be performed reactively.

## 2. Methods

### 2.1. Participants

Forty participants (mean age 23.33 ± 3.66 yrs., 25 women, 15 men) joined this experiment. One participant declined to give their age. Participants received financial compensation or course credits for their efforts. The experiment was conducted in agreement with the Declaration of Helsinki (2013, except pre-registration of the study) and was approved by the local ethics committee of the Faculty of Psychology and Sports Science at the Justus Liebig University Giessen, Germany. Before taking part in the experiment, all participants provided written consent. All participants were naïve as to the purpose of the study at the time of testing. They reported normal or corrected-to-normal vision and that they were right-handed. Participants reported no vestibular, balance, hearing, or depth perception problems, and no problems with feeling in their hands.

### 2.2. Apparatus

An HTC Vive Pro Eye virtual head-mounted-display (HMD; HTC Corp., Xindian, New Taipei, Taiwan) was used to present the visual stimuli. The HMD has a field of view that extends approximately ±110° diagonally. The HMD screen has a 1400 × 1600 pixel resolution per eye and a 90 Hz refresh rate. Participants held an HTC Vive Pro controller in their right hand, hereafter simply referred to as VR controller, through which we recorded the position of the participant's hand at 90 Hz. We used the integrated haptic actuator of this controller to present the vibrotactile probe stimuli on each participant's hand. Visual stimuli were created in Unity (version 2019.4.16f1; Unity Technologies Inc., San Francisco, CA, USA) and displayed via SteamVR (version 1.20.1). The experiment was run on an Alienware desktop PC (Intel Core i9-7980XE CPU at 2.6 GHz, 32 GB RAM, Dual NVidia GeForce GTX1080 Ti GPU). The projection was stereoscopic and was actively linked to the position of the participant's head. Therefore, distance cues were available from stereoscopic cues and motion parallax. Auditory pink noise was played over the HMD's headphones to ensure that participants based their responses during the tactile detection task only on the tactile perception from the vibrotactile stimuli and not from possible audible noise arising from the haptic actuator.

### 2.3. Visual and tactile stimuli

#### 2.3.1. Visual environment

**Figure 1.**
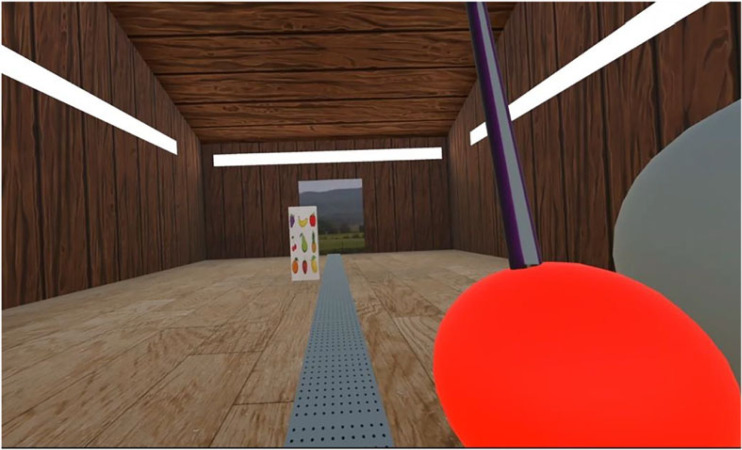
A depiction of the virtual barn environment. The figure shows the upright object to be interacted with, approaching the participant from the far end of the barn. Three of the strip lights are visible on the walls and the grey checkered line on the floor. The red and grey spheres appear on the side of the moving right arm. To start the trial, participants had to place the virtual laser sword, shown with a coloured texture applied, within the red sphere, and they were required to intercept the target object only after first moving towards the grey sphere located right of the red sphere to increase the movement amplitude and consistency across trials and participants.

Participants viewed a virtual barn environment that had a 20 × 30 m floor, as well as four walls and a steepled ceiling that was 13 m tall at the highest point. The surfaces had a brown wood texture. Each wall had a panel strip light that was 18 m long and 1 m wide. There was one additional strip light on the highest point of the ceiling. At the end of the barn, opposite the participant, was a picture of a field from the German countryside that was 5 m long and 2.5 m wide. This functioned as the door from where the target objects would appear. On the floor, where the participant stood, there was a 1 m wide grey checked line that extended down the centre of the barn to the door. There were no shadows in the environment. A screenshot depicting this environment is presented in figure 1.

#### 2.3.2. Virtual objects

The VR controller that the participants held in their right hand was depicted in the virtual environment as it was in the real world except that it was black instead of blue. Attached to the virtual VR controller was a virtual cylinder (0.05 m diameter, 0.56 m tall) that appeared as a laser sword. The laser sword was attached to the virtual VR controller so that when participants moved their hand the laser sword moved with it. The bottom of the laser sword was at the location of the VR controller.

A rectangular object (0.5 m wide × 1.5 m long × 0.3 m in depth), hereafter referred to as *target object*, could be presented in two possible initial orientations: either ‘Upright’ such that its long axis was orthogonal to the floor, or ‘Rotated’ such that its long axis was parallel to the floor. The object could have either a black and white diagonal striped texture, or a texture that contained different fruits for the practice and experimental trials, respectively (for details please see below).

Two spheres were projected in front of where the participant had to stand; a red sphere (diameter of 4 m) that was 2 m above the ground and appeared right in front of the participant. The red sphere was used as a starting position for the virtual sword in each trial. The target object's initial position was 23 m in front of the red sphere. In addition, a grey sphere (diameter of 0.6 m) that was 2.2 m above the ground appeared 0.4 m to the right of the centre of the red sphere and 0.1 m closer to the participant. The grey sphere appeared only during movement trials, when a target object would have to be intercepted at a position to the left of the red sphere. This grey sphere was used to encourage participants to make an initial movement away from the target object and subsequently, perform a larger movement to intercept the target object. With this procedure, we ensured that the movement was long enough to allow the target object to rotate and then present the vibration stimulus 100 ms afterwards, while allowing for a comfortable starting posture of the participant's arm.

Participants reported whether or not they felt the vibration stimulus presented through the VR controller by hitting with their virtual sword a ‘Ja’ (Yes) or ‘Nein’ (No) virtual cube which appeared at the end of each trial. Each cube was 0.4 m^3^ in size and located 1.7 m above the ground. The ‘Ja’ and ‘Nein’ cubes were located 0.5 m to the left and to the right of the centre of the grey checked line, respectively. The configuration of the possible responses was fixed across participants.

#### 2.3.3. Tactile stimuli

In order to probe tactile sensitivity, we delivered brief vibrations to the palm of the right hand via the VR controller [24]. The vibration was presented at 250 Hz for 50 ms at one of eight possible intensities (0.00099, 0.0033, 0.0066, 0.0099, 0.033, 0.066, 0.099, 0.33 arbitrary units) plus a no-vibration condition. For each task, each intensity, including the no-vibration condition, was presented five times. The intensities provided by the VR controller do not correspond to a physical vibration amplitude such as a peak-to-peak displacement and are simply defined by an arbitrary number between 0 and 1. In a pilot study, we found that all intensities below 0.0009 units lead to the same mechanical output, likely due to the limitations of the haptic actuator, and therefore this intensity was the lowest one that we chose to present.

### 2.4. Task and experimental conditions

Participants performed a dynamic object interaction task in which they had to intercept a target object approaching at a constant speed of 5 m s^−1^ with a virtual sword. In addition, they performed a tactile detection task in which they had to detect a vibrotactile stimulus presented during the trial (see Procedure). The experiment consisted of three experimental conditions, that were presented in separate counterbalanced blocks. In the *Predictable Stable* condition, the object always maintained its initial orientation while approaching. In the *Predictable Rotation* condition, the object always appeared in one of the two possible orientations and, when the participant moved their hand towards the target object to intercept it, the target object always switched to the other orientation. In the *Unpredictable Rotation* condition, the object appeared in one of the two possible orientations and, during the participant's interceptive movement, the target object switched to the other orientation in 50% of the trials, whereas in the other 50% of the trials it remained in its initial orientation. In the two *Rotation* conditions, the object switched to the other orientation once the participant's arm movement exceeded a velocity criterion of 0.3 m s^−1^ while moving leftward. In addition to the experimental conditions, we also included a *Baseline* condition presented in a separate block of trials. In this condition, the target object appeared always in the Upright orientation and followed the same trajectory towards the participant without ever rotating. The only difference here was that participants were asked to remain still and perform the tactile detection task. The Baseline condition always occurred first.

Within each of these four blocks, each orientation and possible rotation probability was presented 45 times in a pseudorandomized order. This means that the Predictable Stable and Predictable Rotation conditions included 90 trials (45 trials for each initial object orientation), the Unpredictable Rotation condition included 180 trials (45 trials for each initial object orientation and probability of rotation) and the Baseline task included 45 trials for the single object presented. For each of these conditions, each of the nine tactile probe intensities was presented five times in a pseudorandomized order.

### 2.5. Task load

Task load was assessed using the NASA Task Load Index [28–30]. We used the German translation [31] (see also [29]) which can be retrieved from (http://interaction-design-group.de/toolbox/wp-content/uploads/2016/05/NASA-TLX.pdf) The NASA Task Load Index consists of six subscales which measure different aspects of task demand: Mental Demand, Physical Demand, Temporal Demand, Effort, Performance and Frustration Load. A description of each subscale is provided next to it. Each subscale shows a box made of 20 vertical lines. The participant marked the line they felt corresponded best to their experienced task load. Each participant assessed task load at the end of the experiment separately for each of the four conditions. We calculated a Global Task Load Score across all subscales for each condition ranging between 0% and 100% with high numbers indicating high task load [28,29].

### 2.6. Procedure

**Figure 2.**
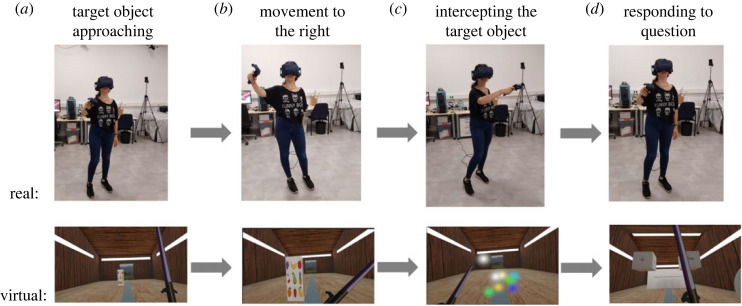
Trial schedule. The top row shows the participant and the bottom row the virtual scene inside the HMD. (*a*) *Target Object Approaching* shows the participant in the real world just after the red sphere had turned off and the participant is free to move. In the virtual environment, we can see the barn environment with the target object approaching the participant. (*b*) *Movement To The Right* shows the participant moving their arm (laser sword in VR) towards the not pictured, grey sphere on their right. (*c*) *Intercepting the Target Object* shows the participant moving their hand/laser sword leftward to intercept the target object. This panel depicts the participant just after they have successfully interacted with the object, turning the object into sparkles to denote the successful completion of the trial. (*d*) *Responding To Question* shows the participant looking at the response cubes and making a movement with the virtual sword towards one of the response cubes (yes or no) to indicate if they had felt a vibration on their moving hand.

The procedure is illustrated in figure 2, additionally a video of two trials of the Unpredictable Rotation condition can be found at: https://doi.org/10.17605/OSF.IO/G5CPQ.

After arriving in the laboratory participants were instructed about the task and asked to sign an informed consent form. Once signed, a more detailed set of instructions were given including how to hold the controller and put on the HMD so that it felt comfortable. After placing the HMD on their head and adjusting the interpupillary distance, the experiment started. Participants were instructed to stand on the grey checkered line on the floor and viewed a series of instructions that were projected into the VR environment. After they had finished reading the instructions, the virtual laser sword appeared attached to the virtual VR controller. The grey and red sphere also appeared in the barn and participants were asked to adjust their distance to the red sphere so that the base of the laser sword could be comfortably placed into it and moved into the grey sphere. Participants could adjust their standing position forwards or backwards so long as they remained on the grey checkered line and kept the red sphere in front of them. This allowed participants of different heights to complete the task comfortably. The pink noise was turned on at this point in order to mask the sound of the vibration produced by the VR controllers. Participants then viewed another set of instructions explaining that they were going to start the practice trials for the baseline condition, which was always presented first. This set of instructions also informed participants that they would not receive vibrations during the practice trials. This was done so that participants could focus on learning the proper movement without having to perform a dual task. Participants completed 24 practice trials prior to each experimental condition, and 12 practice trials for the baseline condition.

After completing the practice trials another set of instructions appeared informing them about the start of the baseline condition and the additional performance of the vibration detection task. After the completion of the baseline condition, the experimental conditions proceeded similar to the above-mentioned procedure. The order of the three experimental conditions was counterbalanced across participants. Participants were allowed to take a break at any time, however if they wanted to remove the HMD for a longer break, they were encouraged to wait until finishing the current block. Once all conditions were completed, participants completed the NASA Task Load questionnaires, one for each condition. The experiment took approximately 1 ½ - 2 h.

Each trial started by the participant putting their hand into the red sphere and clicking the trigger button of the VR controller. This caused the target object to appear in front of the participant. When travelling towards the participant, the furthest right side of the object was positioned just to the left of the grey checkered line (figure 1). Participants were instructed to successfully intercept it with their virtual sword by hitting the object's rightmost side. To avoid stereotyped movements towards the object's centre and prompt for online adjustments of the movement, the centre of the rotated object appeared 1.25 m higher than the centre of the object in the upright orientation, so that there was a spatial gap of 0.25 m between the highest edge of the upright object and the lowest edge of the rotated object. This means that when the target object rotated, the participants would have to adjust their movement by a minimum of 0.25 m in the vertical direction to ensure successful object interception.

At the start of a trial in the experimental conditions, the participant placed the base of the virtual sword within the red sphere and kept it there as long as the red sphere was visible. Once the target object was 6 m away from the red sphere, the red sphere disappeared and the participant was free to move the virtual sword. If they moved outside of the red sphere before it disappeared the target object was teleported back to the far end of the barn. Once the participant placed the hand back in the red sphere the trial started again. After the red sphere disappeared the participant first had to move their arm rightward into the large grey sphere and then leftward to intercept the target object. When the participant was moving leftward at more than 0.3 m s^−1^ and the target object was within 3 m of the VR controller, the target object could rotate from its current orientation by 90° in a clockwise direction (a roll rotation), and a probe vibrotactile stimulus was presented 100 ms after that moment. Please note that this moment occurs independently of whether the object actually rotated or not. Therefore, the probe stimuli were presented both in rotation and no-rotation trials at the same moment during the movement. This probing moment happened *at least* 100 ms prior to intercepting the object. If the participant successfully intercepted the object, the object exploded and colourful virtual sparkles appeared. If the participant was not successful in intercepting the object, the object continued its path until it hit the end of the barn. If a trial was considered a mistrial, the participant repeated this trial right afterwards and the mistrial was not included in the statistical analysis. In the baseline condition, a trial could not be a mistrial as, if the participant left the red sphere, the object would move back to the front of the barn and restart. In the experimental conditions, a trial was considered a mistrial when participants did not enter the grey sphere prior to hitting the object, they did not move faster than 0.3 m s^−1^ during the interceptive movement, they did not intercept the target object before it reached the end of the barn behind the participant (at a distance of 14 m from the centre of the room), or they did not hit the correct side of the object (the rightmost side relative to their view). Since all mistrials were repeated our analysis was based on the same number of trials per condition across participants. In Experiment 1, the median proportion of mistrials per participant was 4.20% (SE = 2.29%), where 8.13% of mistrials occurred in the Predictable Stable condition, 22.09% in the Predictable Rotation and 69.77% in the Unpredictable Rotation condition.

After the object disappeared, two response cubes (yes or no) appeared and the participant had to indicate if they had felt a vibration or not. Participants used the laser sword to touch the respective response cube. After responding, the cubes disappeared and the red sphere reappeared. The participant could then put the laser sword back into the red sphere to start the next trial.

The baseline condition followed the same procedure as the experimental conditions except that the red sphere was always visible and the participant was instructed to never move the laser sword outside of it, except for when responding to the detection task. The target object was approaching the participant as in the experimental conditions; but it was only orientated Upright and never rotated. Once the object was within 3 m of the VR controller the vibration occurred 100 ms later, i.e. to a similar moment as in the experimental conditions, unless it was a no-vibration trial. If the participant moved away from the red sphere, the object was teleported back to the far end of the barn and the trial started again once the participant put their hand back into the red sphere. Once the object reached the far end of the barn, sparkles appeared in front of the participant and the red sphere disappeared. The vibration always occurred at least 100 m prior to the object disappearance. The response cubes were then presented and participants indicated with the sword if they had felt a vibration or not. After responding, the cubes disappeared and the red sphere reappeared. The participant could then put their hand back into the red sphere to start the next trial.

### 2.7. Data analysis

#### 2.7.1. Movement kinematics

During the experiment, on each frame of a trial (every 11 ms) the position of the base of the laser sword, denoting the position of the right hand, was recorded for each participant, trial and condition. We recorded the *x* (left and right), *y* (up and down) and *z* (back and forth) position of the laser sword starting at the onset of the trial and stopped once the target object collided with the sword or a mistrial occurred. As the perturbation of the object was along the *y*-axis, this is where we expect differences in movement kinematics between conditions.

The kinematic data were then analysed using R (R Studio version 2021.09.1). First, all mistrials were removed. Then, for each trial, we determined the median frame on which (a) the vibration of the controller occurred, (b) the rotation of the target object and (c) the collision occurred.

As the perturbation would influence movement kinematics along the vertical dimension, we calculated the average position of the hand along the *y*-axis by collapsing across trials of each condition and initial object orientation, separately for each participant. Thus, for each participant, we obtained in total eight trajectories, two for each of the four conditions (Predictable Stable, Predictable Rotated, Unpredictable-no rotation, Unpredictable- rotation). We then averaged these time courses across the conditions and initial object orientation across the participants.

The interquartile range (IQR) of the hand position was determined similarly for the *y*-axis. We first determined the IQR of the position of the hand for each participant by collapsing across trials, separately for each condition and initial object orientation. Then, using that data, we calculated the overall average IQR of the position of the hand per frame, condition and initial object orientation.

In order to get better insight into how the participants were performing the movement correction and if they were using the changing visual information provided by the different target object orientations, we calculated the movement correction latencies. While different methodologies and analyses can be used, previous studies that have calculated movement correction latencies have focused on the velocity or acceleration of the hand in different conditions. For instance, previous studies have compared differences in hand acceleration following a perturbation upwards or downwards [32], differences in the velocity of grip rotations during object rotation [33], and the velocity of the hand during leftward or rightward perturbations compared to an unperturbed condition [34]. Here, we compared the velocity of the hand in our perturbation conditions with that in our no perturbation conditions. Velocity was used as the measure of latency instead of acceleration as the averaged acceleration correction latencies tend to be overestimated and less precise [35]. Additionally, we focused only on the Predictable Stable, Predictable Rotation and Unpredictable Rotation conditions, when the object actually rotated and thus movement corrections could be detected.

With these conditions, we could compare the latency of the movement correction to the Predictable Stable condition where no movement correction was expected (see [34,36,37] for other studies which have compared a perturbation condition to a no-perturbation condition). To compare the rotation conditions to the Predictable Stable condition, we grouped the conditions with respect to the initial orientation of the target object. First, we calculated the average velocity of the hand on the *y*-axis for each participant, condition, frame and initial orientation of the target object (i.e. we collapsed across trial). We then truncated the average data of each participant and condition from the overall average time of the rotation of the target object. Using this data, we ran two separate linear mixed models to examine at which point, relative to perturbation onset, the rotation conditions started to differ from the Predictable Stable condition, separately for the Upright and Rotated target object orientations. The time at which the rotation conditions started to differ from the Predictable Stable condition was taken as the correction latency.

For both linear mixed models, our dependent variable was the *y*-axis velocity, condition and time were repeated fixed effects, and participant was a random effect. Multiple comparisons were corrected with a Sidak correction.

#### 2.7.2. Tactile perception

We fit the yes/no responses of the tactile detection task to nine separate psychometric functions (1 for Baseline, 2 for Predictable Stable (Upright object and Rotated object), 2 for Predictable Rotation (the initially Upright object that always rotates from Upright to Rotated, and the initially Rotated object that always rotates from Rotated to Upright) and 4 for Unpredictable Rotation (all combinations). This was done to investigate if there were any differences in the detection thresholds within each of the experimental conditions. Because the Rotated object had a smaller surface area than the Upright object, it was potentially harder to interact with, which could have potentially affected the detection threshold. The data was fit using the R (RStudio Version 1.4.1717) package *quickpsy* [38], using a Weibull distribution and optimized via the Differential Evolution algorithm [39]. This algorithm requires upper and lower bounds for the parameters. We chose the upper and lower vibration capabilities of the VR Controller for the means, which were 0 and 1, and 0 and 2 for the standard deviations, which corresponds to well-above any values expected from the detection thresholds. The 50% threshold of each psychometric function was used to determine the vibration intensity where participants had a 50% chance in detecting the vibration (detection threshold).

An outlier analysis was then run on the thresholds for each task. A data point was removed if it was ± 2.5 s.d. away from the mean across the thresholds of this condition. This resulted in the removal of 30 out of 360 thresholds (9 thresholds×40 participants; 8.3% of the data).

As detection thresholds did not differ for the different object rotations within each condition (*p* > 0.05 in all cases), we collapsed the data and recalculated the thresholds for each participant, one for each condition (Baseline, Predictable Stable, Predictable Rotation, Unpredictable Rotation). The false alarm rate was also calculated for each condition as well as the overall average false alarm rate (false alarm rates were similar across all conditions). The overall false alarm rate was determined by averaging the false alarm rate across participants per condition.

To assess possible effects of the predictability of rotation on tactile processing and on task load in the detection threshold in each of the four tasks, we employed three separate linear mixed models with *condition* being set as a fixed repeated effect and *participant* as a random effect (random intercept) which allowed us to take into account the variability in performance between participants. The first linear mixed model included all conditions and investigated possible effects of movement on tactile suppression. The second linear mixed model was performed without the baseline condition because it examined the effect of condition (predictability) on the thresholds by controlling for differences in movement speed. Therefore, movement speed (speed of the hand on vibration) was added as a covariate. The third linear mixed model examined the effect of condition (predictability) on Task Load.

Degrees of freedom were approximated using the Satterthwaite method [40]. *Post hoc* pairwise comparisons were made using the Sidak correction. The alpha value displayed below is an adjusted value that has been corrected for the multiple comparisons within a given *post hoc* test using the Sidak correction, such that a ‘corrected alpha’ of less than 0.05 indicates statistical significance. The amount of correction is based on the number of comparisons made. The assumptions of the normality, homoscedasticity and multicollinearity of the residuals were checked for each linear mixed model. There were some minor deviations from normality for the threshold data, which given the robustness of linear mixed models [41] and the size of the sample should not affect the interpretation of the results. All data used in the experiment and code is available at https://doi.org/10.17605/OSF.IO/G5CPQ.

## 3. Results

### 3.1. Movement kinematics and timing

**Figure 3.**
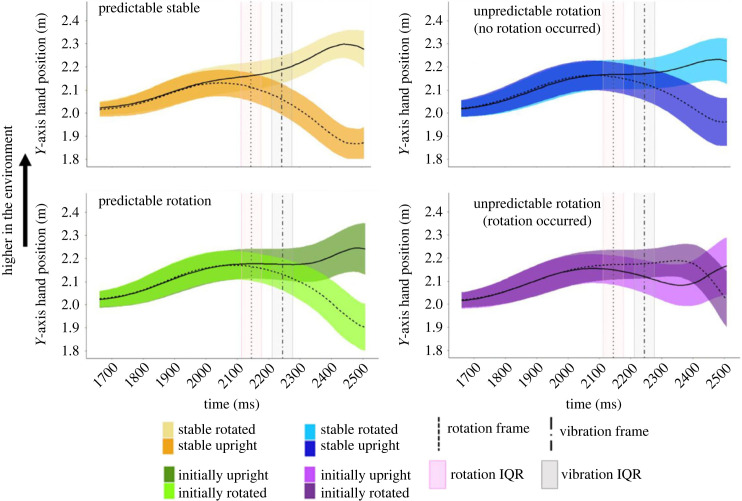
Average hand position for each condition along the vertical axis (*Y*-axis). The median time at which participants collided with the object was around 2508 ms into the trial. The higher the line, the higher the hand's position in the virtual environment. The horizontal shaded areas represent the ± standard deviation around the mean hand position across all participants and trials for a given condition. The median time at which the target object was triggered to rotate and when the controller could have vibrated are indicated in the figures as the dotted line and the dot dash line, respectively. The pink vertical shaded area corresponds to the interquartile range around the median time the object was triggered to rotate. The grey vertical shaded area corresponds to the interquartile range around the median time the controller vibrated. To compare the data between rotating and not rotating trials, the median time where the object was triggered to rotate, along with its interquartile range, was included in all figures even if a rotation did not occur in that condition.

The average *y*-axis hand position per task is plotted in figure 3 for every 11 ms (one frame) during a trial. The median time where the target object rotated was 2145 ms (s.d. = 64.79 ms) from the start of the trial and the median time the controller vibrated was 2244 ms (s.d. = 64.79 ms) from the start of the trial. The median time where participants collided with the object was 2508 ms (IQR = 70.84 ms) from the start of the trial.

When participants were moving along the *y*-axis in the environment, their movement trajectories followed a similar pattern when the target object rotation was predictable or when the object did not rotate (figure 3): in these conditions, participants started their movement from roughly midway between both objects and then moved up if the object was always horizontal (yellow trace), or initially vertical and then always rotated to horizontal (dark green trace). Likewise, they moved downward if the object was always vertical (orange trace), or initially horizontal and then always rotated to vertical (light green trace). As shown in figure 3, the timing of the movement is slightly delayed in the Predictable Rotation and Unpredictable Rotation (no rotation) conditions compared to the Predictable Stable condition. However, for the Unpredictable Rotation condition, we see differences in the average movement trajectory compared to the stable or predictable conditions. Participants' initial movement trajectory followed closely to the Unpredictable Rotation (no rotation) condition until *ca* 2350 ms, approximately 200 ms after the unexpected rotation of the object, where they then correct their movement trajectory and quickly move to intercept the target object at its new location.

### 3.2. Movement correction latency

**Figure 4.**
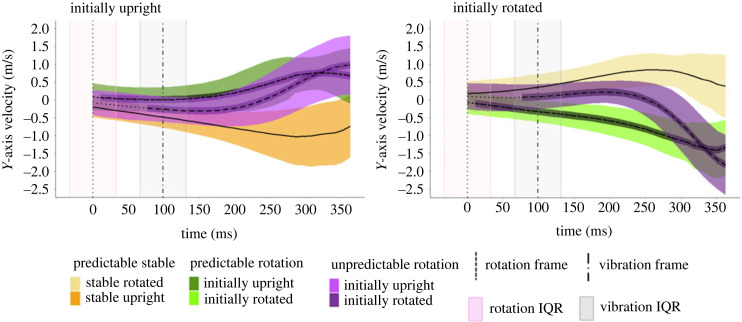
The average velocity of the hand on the *y*-axis for the different rotation conditions compared to the Predictable Stable condition. The figures are split up depending on the initial orientation of the target object. Positive numbers indicate that the hand was moving upwards. The horizontal shaded areas represent the ± standard deviation around the mean hand velocity across all participants for a given condition. The narrow dark horizontal area around the velocity line in the rotation conditions indicates that the given condition differs from the Predictable Stable condition at that time. The median time at which the target object was triggered to rotate and when the controller could have vibrated are indicated in the figures as the dotted line and the dot dash line, respectively. The vertical shaded areas correspond to the interquartile range around the median time of the respective event.

There was a significant interaction between time and condition for the linear mixed model that compared the Initially Upright target objects (*F*_66, 152.248_ = 13.79, *p* < 0.001) as well as for the linear mixed model that compared the Initially Rotated target objects (*F*_66, 138.844_ = 23.276, *p* < 0.001) figure 4.

The latencies were determined in both linear mixed models and then averaged across the two object orientations for each condition. The latency in the Predictable Rotation condition was just 11 ms (being identical for both the initially upright and initially rotated target objects), indicating that participants had already started deviating their hand trajectory to successfully hit the object at its anticipated target position, even before the object rotated. The latency in the Unpredictable Rotation condition was unsurprisingly longer (77 ms for both the Initially Upright and Initially Rotated target objects). In all cases, once a rotation condition started to differ from the Predictable Stable condition, it remained significantly different until the end of the trial.

### 3.3. Tactile suppression

We found a main effect of condition on detection thresholds (*F*_3, 54.75_ = 18.36, *p* < 0.001). Pairwise comparisons revealed that all conditions differed from baseline (all *p* < 0.001, table 1), demonstrating that movement led to tactile suppression, in line with previous findings (e.g. [2]). No other significant effects were found (all *p* > 0.05). The overall false alarm rate was 4.87% (s.d. = 12.25) across conditions (Baseline: 5.25%, s.d. = 14.67, Predictable Stable 5.75%, s.d. = 12.06, Predictable Rotation 4.06%, s.d. = 11.78, Unpredictable Rotation 4.41%, s.d. = 10.46).

**Table 1.** The mean differences in tactile detection thresholds between the Baseline and the experimental conditions. SE, standard error of the mean; Sig., significance.

(I) condition	(J) condition	mean difference (I-J)	SE	Sig.	95% confidence interval for difference	
					lower bound	upper bound
baseline	Predictable Stable	−0.067	.016	0.001	−0.111	−0.022
	Predictable Rotation	−0.102	.024	<0.001	−0.167	−0.037
	Unpredictable Rotation	−0.138	.029	<0.001	−0.219	−0.057

### 3.4. The effect of predictability on detection thresholds

**Figure 5.**
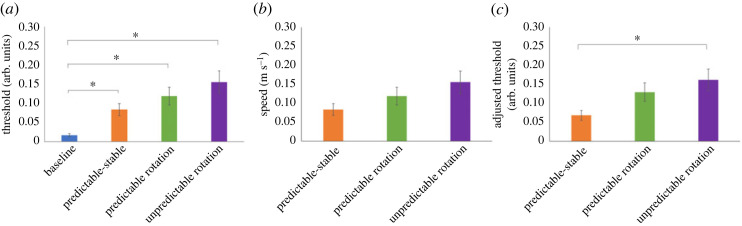
Average raw detection thresholds (*a*), the three-dimensional speed of the hand at the time of vibration (*b*), and the average adjusted detection thresholds (*c*), plotted for each condition. The adjusted thresholds (*c*) for each condition are the estimated marginal means and so have been adjusted for the speed of the participant's hand at the time of vibration during that task. Error bars are ± standard errors of the mean. The asterisks indicate that the conditions differ significantly (*p* < 0.05).

There is evidence that movement speed could affect the strength of suppression with faster speeds leading to higher detection thresholds [16], but see also [14]. We ran a separate liner mixed model to determine if the speed of the hand at the time of vibration might have an effect on the experimental conditions. The speed of the hand was taken as the dependent variable while condition was set as a fixed repeated effect. Participant was used as a random variable (Intercept). The baseline condition was not included as participants had been instructed not to move. No significant effects of condition were found (*F*_2, 72.804_ = 2.37, *p* = 0.100). To keep a naturalistic movement behaviour, we did not constrain participants’ movement preferences, and thus different participants may have moved at different speeds, including the time of presenting the probe stimulus, which, based on previous literature, could affect tactile suppression [16]. To account for possible effects of movement speed on tactile suppression, we rerun the linear mixed model with the instantaneous three-dimensional speed of the hand at the moment of vibration (averaged across trials of the given condition) as a covariate for the experimental conditions (figure 5). For this analysis, we did not consider the Baseline condition as participants did not move and thus no hand speed could be determined. We again found a main effect of condition on detection thresholds (*F*_2, 60.64_ = 5.77, *p =* 0.005), as reported earlier. In contrast to the raw detection thresholds analysis reported before, *post hoc* pairwise comparisons revealed that tactile suppression in the Predictable Stable condition was lower than in the Unpredictable Rotation condition (mean difference Predictable Stable minus Unpredictable Rotation = −0.092, *SE* = 0.031, *p* = 0.013, CI = −0.169 – (−0.016)). No other effects were found (*p* > 0.05).

### 3.5. Task load

**Figure 6.**
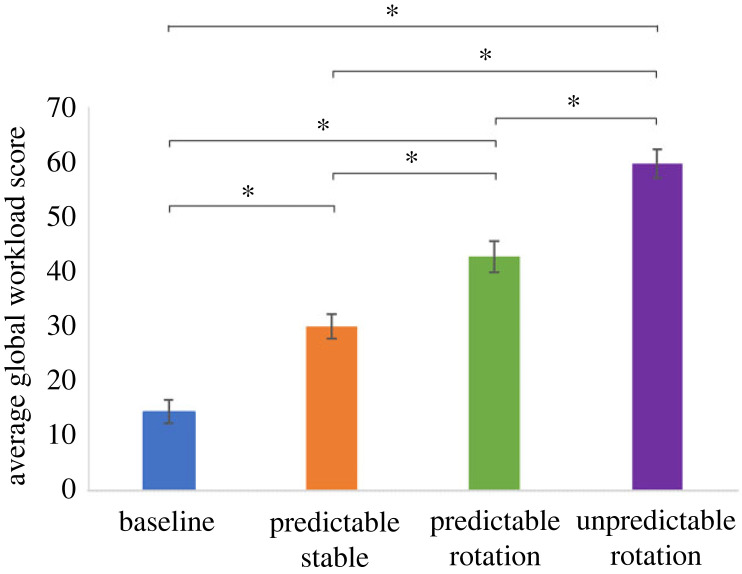
The mean Global Task Load Score for each condition for experiment 1. Error bars are ± standard errors of the mean. The asterisks indicate that the conditions differ significantly (*p* < 0.05).

The Global Task Load Score was calculated from the subscales for each condition for each participant (figure 6). There was a significant main effect of condition (*F*_3, 71.316_ = 65.18, *p* < 0.001). Pairwise comparisons revealed that all conditions differed from each other (all *p* < 0.003, table 2).

**Table 2.** The mean differences in Global Task Load Score between the conditions. SE, standard error of the mean; Sig., significance.

(I) condition	(J) condition	mean difference (I-J)	SE	Sig.	95% confidence interval for difference	
					lower bound	upper bound
baseline	Predictable Stable	−15.750	3.128	<0.001	−24.194	−7.306
	Predictable Rotation	−28.458	3.454	<0.001	−37.796	−19.121
	Unpredictable Rotation	−45.567	3.383	<0.001	−54.709	−36.425
Predictable Stable	Predictable Rotation	−12.708	3.536	0.003	−22.260	−3.157
	Unpredictable Rotation	−29.817	3.466	<0.001	−39.178	−20.455
Predictable Rotation	Unpredictable Rotation	−17.108	3.763	<0.001	−27.268	−6.949

## 4. Discussion

Tactile suppression has primarily been examined in small-scale movements, such as finger extension and flexion [9,42] or goal-directed movements in stationary environments, such as reaching to [5] or grasping an object [3,6,22]. Here, we replicated the classical tactile suppression effect in a dynamic object interaction task performed in a complex visual environment. To our knowledge, this is the first experiment to do so, though see also [6]. Further, this is the first tactile suppression experiment which examined changes in tactile sensitivity under varying levels of visuomotor predictability allowing for preplanned instead of reactive movement adjustments if the object's behaviour was predictable.

Participants’ movement trajectories showed that they were able to successfully adjust their movements to the behaviour of the target object by aiming at different locations depending on the object's future orientation. For the Unpredictable Rotation condition, we observed a delay in participants' vertical movements after the object's rotation. It is likely that participants initially aimed for the target object at its original height and then recalculated their movement with respect to the new orientation resulting in the temporal lag. Such a delay was not present in the Predictable Rotation condition where the required adjustment of the movement could be preplanned. This delay is further supported by our analysis on the movement correction latencies which showed that overall, when the rotation was predictable, the correction latency occurred sooner than if that rotation was not predictable.

Previous studies have shown that reliable sensorimotor predictions can lead to an increase in the strength of tactile suppression [8,9,22]. We hypothesized that if participants had to change their movement trajectory in response to an unexpected perturbation of the target object, there should be a decrease in the tactile suppression relative to the predictable conditions, which might reflect facilitated somatosensory feedback processing on the moving hand. This should lead to a decrease in the tactile detection thresholds in the unpredictable compared to the predictable condition. Instead, we found that whenever the participant had to change their movement trajectory unpredictably, there was an *increase* in suppression compared to when the participants' movement was predictable. Such an increase in threshold is likely related to the higher task load caused by the unexpected adjustments of the movement trajectory, as supported by higher Global Task Load scores. This is in line with the high number of mistrials in the Unpredictable Rotation condition, which in turn led to longer blocks of trials (mistrials were repeated at the end of the block). As recent work does not provide evidence for a change of tactile suppression over time, at least within a window of 20 min [8], it seems to be rather unlikely that a longer block duration would have affected the strength of tactile suppression. Higher cognitive load has been associated with reduced executive capacities and increased tactile suppression [43]. In more demanding tasks, participants might have withdrawn processing resources from the tactile detection task in order to shift more resources to the more challenging movement task leading to an increase in sensory threshold. If an increase in task load is related to an increase in tactile suppression, this would support the idea that tactile suppression frees up cognitive resources to process task relevant sensory information [42,44]. However, although Global Task Load scores for the Predictable Rotation condition were significantly higher than those in the Predictable Stable condition and significantly lower than those in the Unpredictable Rotation condition, the resulting thresholds did not differ significantly from either other despite a significant main effect of condition (figure 5*c*), which is presumably due to an overall increase in task load in the rotation conditions.

Participants could have also performed the task by relying on visual cues only which might have further reduced the processing of somatosensory input from the moving hand. Due to the nature of VR, participants could not see their real hand. However, they saw a virtual laser sword in the same location which would have likely led to an increased reliance on visual cues to determine the position of the hand. Indeed, when interacting with visual targets, people rely more on vision than on somatosensation [20]. Similarly, if participants have to make unexpected movement corrections in response to the target's perturbation, they might have to rely even more on the visual cues to determine the position of their hand. Following this argumentation, removing the visual feedback of the hand should result in a higher weight given to somatosensory cues [45,46]. Accordingly, a higher weight on somatosensation might reduce tactile suppression and lead to a decrease in detection thresholds, particularly in the Unpredictable Rotation condition. To test this, we designed a follow-up experiment in which we aimed to increase the reliance on somatosensory input from the moving hand by making the virtual laser sword invisible when the target object approached the participant. If this reliance influences tactile suppression, then we should observe lower tactile suppression in the unpredictable compared to the predictable condition as participants will have to rely even more on somatosensory feedback to ensure successful interaction with the target object. However, there is evidence that removing visual feedback of the moving hand leads to stronger suppression [23,47,48] and thus to an increase in tactile suppression.

## 5. Experiment 2: introduction

The results from Experiment 1 showed that when participants cannot plan an upcoming corrective movement, they have higher tactile detection thresholds compared to when they can. The Unpredictable Rotation condition had both the highest thresholds and highest Global Task Load scores. It is possible that the higher detection thresholds might have resulted in withdrawing processing resources from the tactile detection task [43]. Moreover, the movement task could be performed by relying on visual cues only as we provided visual feedback of both the location of the target object and the hand represented by the virtual sword. In Experiment 2, we aimed to increase the weight given to the somatosensory cues of the moving hand by removing the visual feedback of the hand [45] with the aim of examining possible differences in detection thresholds between the conditions. To this end, the virtual sword was rendered invisible during movement towards the target object. A new sample of participants performed the Predictable Stable and the Unpredictable Rotation condition, which showed the most pronounced differences in detection thresholds in Experiment 1, as well as the Baseline condition.

## 6. Methods

A total of 34 new participants (mean age 23.29 ± 2.80 yrs, 26 women, 8 men) joined Experiment 2. In the following, we only report the methods that differ from Experiment 1; all other factors were the same as Experiment 1.

When the object was 12 m from the participant, the virtual laser sword and virtual VR controller turned invisible until either the participant had successfully interacted with the object or the trial was determined to be a mistrial. Considering that the object moved with a constant speed of 5 m s^−1^, and that participants could intercept the object at the earliest when it was 3 m away from them, the virtual sword was invisible for at least 1.8 s in each trial. When the question regarding the detection of the vibration appeared, the virtual laser sword became visible again. No other changes were made.

For the outlier analysis (see Experiment 1), 20 data points out of 238 were removed (8.4% excluded data points). The median mistrial amount per participant was 14.06% (SE = 6.65%), with 15.74% of those trials occurring in the Predictable Stable condition and 84.26% occurring in the Unpredictable Rotation condition. The higher per cent of mistrials per participant in Experiment 2 compared to Experiment 1 is likely due to the removal of the visual feedback of the virtual sword during movement. Additionally, because the Predictable Rotation condition of Experiment 1 is not included in Experiment 2, the contribution of mistrials in the remaining conditions in Experiment 2 is inflated.

In order to investigate if there was a relationship between tactile suppression and task load three correlations were run between the Global Task Load scores and the un-covaried detection thresholds. Firstly, we compared the relationship between the tactile detection threshold and Global Task Load score within Experiment 1 and Experiment 2. For each experiment, we used all of the data from all of the conditions. We then combined the data of the two experiments into one dataset, excluding the 2 baseline conditions and the Predictable Rotation condition (only ran in Experiment 1), and ran a correlation between the tactile detection thresholds and their corresponding Global Task Load scores. We did not include the baseline conditions here as we were interested in the effect of task load on movement-related tactile suppression.

## 7. Results

### 7.1. Movement kinematics and timing

**Figure 7.**
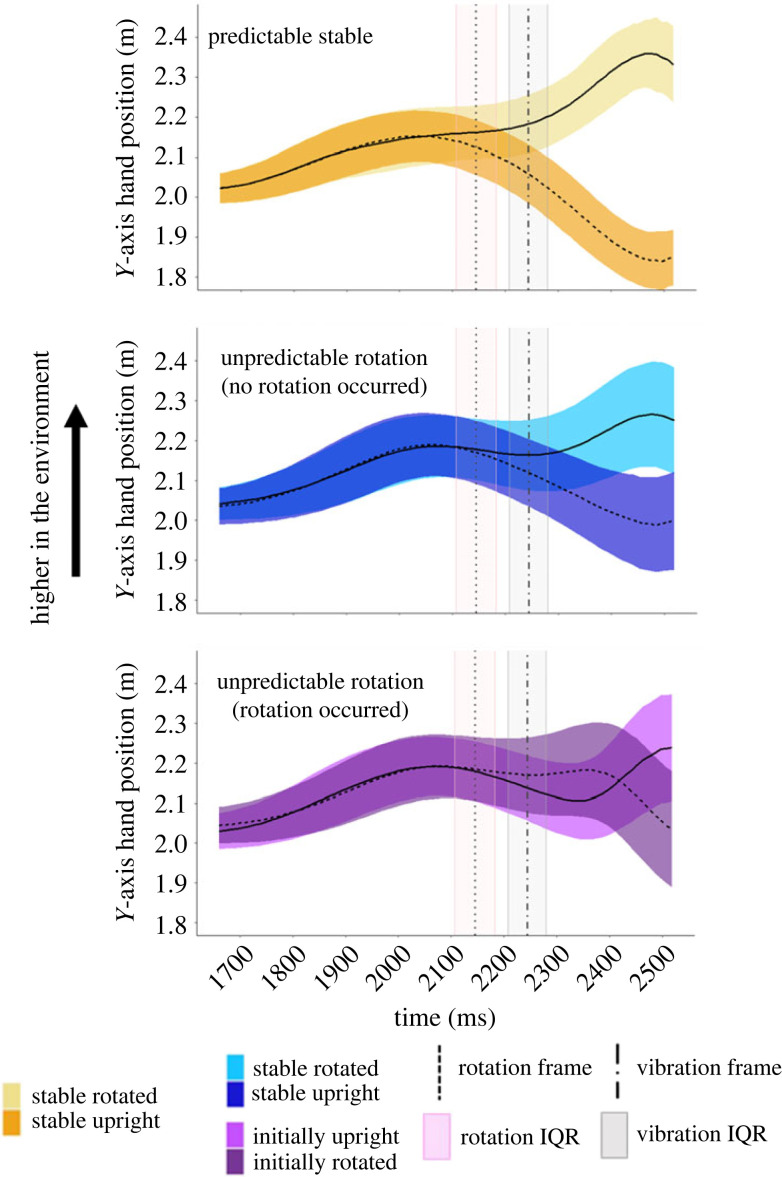
Average hand position along the *Y*-axis for a trial for each condition. Details as in figure 3.

The average hand position time course per task is plotted in figure 7. Relative to the start of the trial, the median time was 2145 ms (IQR = 75.46) for the object rotation, 2244 ms (IQR = 72.27) for the controller vibration and 2530 ms (IQR = 94.27 ms) for the collision with the target object.

When participants were moving along the *y*-axis in the environment, they started their movement from roughly midway between both objects (figure 7). Then they moved up if the object was always horizontal (yellow trace). Similarly, they moved downward if the object was always vertical (orange trace). As in Experiment 1, the timing of the movement up or down is slightly delayed in the Unpredictable Rotation condition compared to the Predictable Stable condition. For the Unpredictable Rotation condition there are also differences in the average movement trajectory between the no rotation and rotation conditions. Participants' initial movement trajectory in the Unpredictable Rotation (rotation) condition followed closely the Unpredictable Rotation (no rotation) condition until *ca* 2350 ms, approximately 200 ms after the unexpected rotation of the object, where they then corrected their movement trajectory and quickly moved to intercept the target object at its new location.

### 7.2. Movement correction latency

We calculated the movement correction latencies for both initial orientations of the target object as well as running two additional linear mixed models, as in Experiment 1, with the exception that the Predictable Rotation condition could not be included.

**Figure 8.**
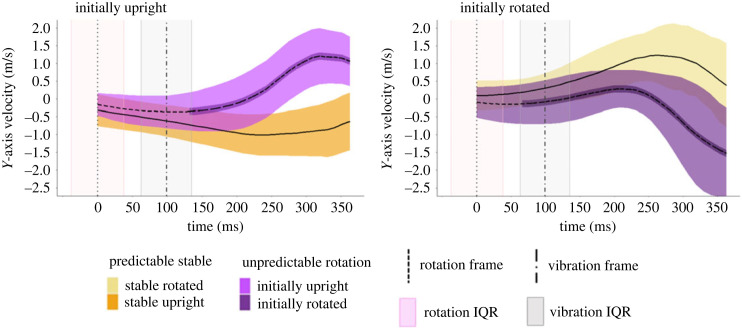
The average velocity of the hand on the *y*-axis for the different rotation conditions compared to the Predictable Stable condition. Details as in figure 4.

There was a significant interaction between time and condition for the Initially Upright target object linear mixed model (*F*_5, 63.705_ = 11.85, *p* < 0.001) as well as for the Initially Rotated target object linear mixed model (*F*_35, 62.014_ = 5.00, *p* < 0.001) figure 8.

The latencies were determined for the Unpredictable Rotation condition for both orientations of the target object and then averaged. The Unpredictable Rotation condition started to differ from the Predictable Stable condition 110 ms after the start of the rotation (it was 194 ms for the Initially Upright target object and 77 ms for the Initially Rotated target object). The average latency is in line with previous findings about manual responses to visual perturbations of the target [33,35]. In all cases, once a rotation condition started to differ from the Predictable Stable condition, it remained significantly different until the end of the trial.

### 7.3. Tactile suppression

We found a main effect of condition (*F*_2, 37.44_ = 27.19, *p* < 0.001) on the tactile detection thresholds, with pairwise comparisons revealing that all tasks differed from baseline (all *p* < 0.001, table 3), indicating that movement led to tactile suppression. No other effects were significant (all *p* > 0.05). The overall false alarm rate was 4.35% (s.d. = 10.80) (Baseline 3.53%, s.d. = 11.52; Predictable Stable 4.41%, s.d. = 9.70; Unpredictable Rotation 5.11%, s.d. = 11.18).

**Table 3.** The mean differences between the Baseline condition and other two conditions. SE stands for standard error and Sig. stands for significance.

(I) condition	(J) condition	mean difference (I-J)	SE	Sig.	95% confidence interval for difference	
					lower bound	upper bound
baseline	Predictable Stable	−0.103	0.022	<0.001	−0.159	−0.046
	Unpredictable Rotation	−0.195	0.034	<0.001	−0.280	−0.110

### 7.4. The effect of predictability on detection thresholds

**Figure 9.**
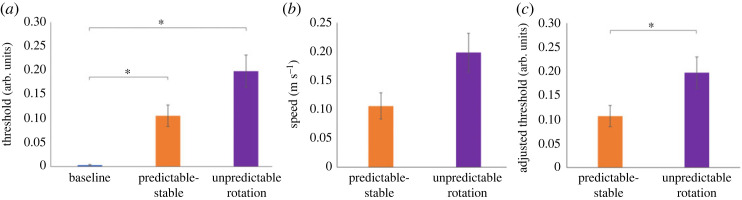
The average detection thresholds (*a*), the average three-dimensional speed of the hand on vibration (*b*) and adjusted thresholds (*c*) for each task. The adjusted thresholds (*c*) for each task have been adjusted for by the speed of the participants’ hand at the time of vibration during that task. Error bars are ± standard errors of the mean. The asterisks indicate that the conditions differ significantly (*p* < 0.05).

As in Experiment 1, there was no significant effect of the condition on the speed of the hand at the time of vibration (*F*_1, 65.123_ = 0.541, *p* = 0.465). When controlling for the speed of the hand at the moment when we presented the tactile probe stimulus, we found a significant main effect of task on detection thresholds (*F*_1, 51.28_ = 5.10, *p =* 0.028) (figure 9), as with the analysis on the raw thresholds. The *post hoc* comparisons now revealed that the two conditions differed, such that suppression was stronger in the Unpredictable than the Predictable condition, in line with the results of Experiment 1 (mean difference Predictable Stable minus Unpredictable Rotation = −0.090, *SE* = 0.040, *p* = 0.028, CI = −0.170 – (−0.010)).

### 7.5. Task load

**Figure 10.**
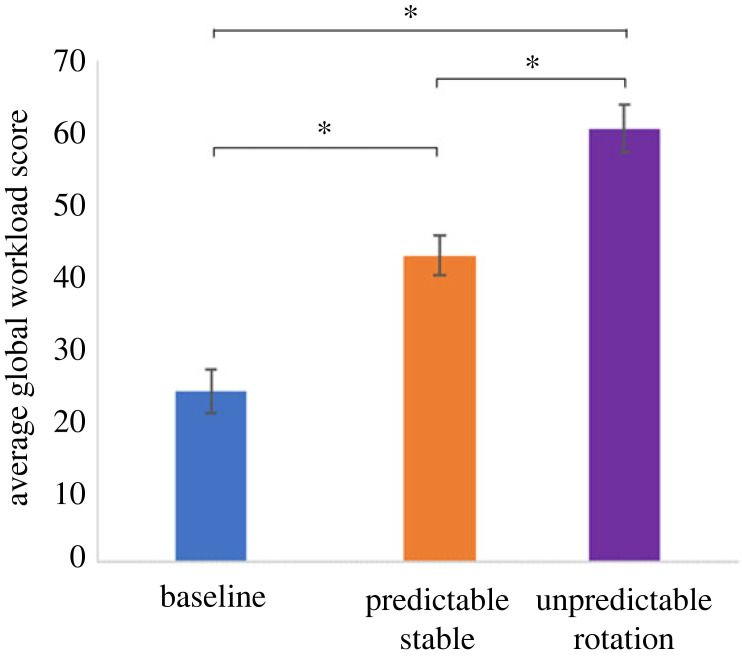
The mean Global Task Load Score for each condition for Experiment 2. Error bars are ± standard errors of the mean. The asterisks indicate that the conditions differ significantly (*p* < 0.05).

There was a significant main effect of condition (*F*_2, 62.21_ = 34.50, *p* < 0.001), with pairwise comparisons revealing that all conditions differed from each other (all *p* < 0.001; figure 10 and table 4). This means that task load was highest in the Unpredictable Rotation condition and lowest in the Baseline, in line with the results of Experiment 1.

**Table 4.** The mean differences between the conditions. SE, standard error of the mean; Sig., significance.

(I) condition	(J) condition	mean difference (I-J)	SE	Sig.	95% confidence interval for difference	
					lower bound	upper bound
Baseline	Predictable Stable	−19.167	4.099	<0.001	−29.211	−9.123
	Unpredictable Rotation	−37.157	4.489	<0.001	−48.156	−26.157
Predictable Stable	Unpredictable Rotation	−17.990	4.338	<0.001	−28.627	−7.353

**Figure 11.**
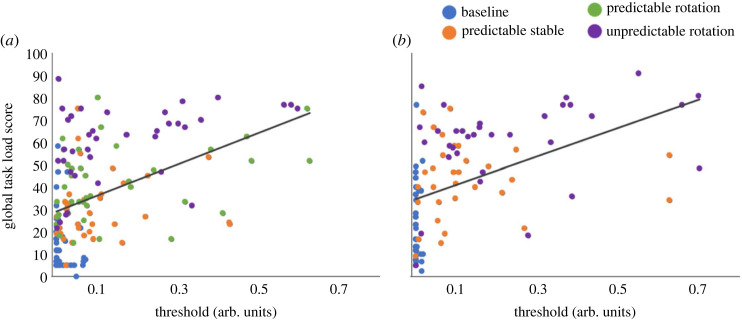
The relationship between detection thresholds and Global Task Load score for both Experiment 1 (*a*) and Experiment 2 (*b*). The regression line was fitted to all data of the respective experiment. Each dot represents one data point (one participant's threshold in a given condition).

We also found significant positive correlations between Global Task Load scores and the detection thresholds for both Experiment 1 (*r* = 0.46, *p* < 0.001) (figure 11*a*) and Experiment 2 (*r* = 0.47, *p* < 0.001) (figure 11*b*), as well as across both experiments (*r* = 0.36, *p* < 0.001), indicating that an increase in Global Task Load is related to an increase in the detection thresholds.

## 8. Discussion

In Experiment 2, we had predicted that removing visual information about the position of the moving hand would lead to increased processing of somatosensory input and thus a stronger decrease in detection threshold in the Unpredictable compared to the Predictable Rotation condition. Such differences might not have been found in Experiment 1 because there, participants might have simply relied mainly on visual information, as is typically the case when performing visually guided hand movements [20]. Thus, by removing visual input about the position of the hand, we sought to increase the reliance on somatosensory feedback control to adjust the movement to the change in object orientation. While we did find a difference in the tactile detection thresholds between the Predictable Stable and the Unpredictable Rotation condition, the Unpredictable Rotation condition continued to have a higher detection threshold instead of a lower detection threshold as hypothesized. Based on an optimal integration model, we would expect that when the weight of one cue, like vision, is decreased, or removed, the reliance on the other senses should flexibly increase [45,49]. This has been found previously for visual and proprioceptive integration; when the gain of the visual cue was manipulated, lowering its reliability, the proprioceptive weighting was higher compared to when the gain of the proprioceptive cue was manipulated [50]. The present results suggest either non-optimal integration or that other factors are driving the effect.

## 9. General discussion

Tactile suppression has been explained as the result of a feedforward mechanism that compares the sensory consequences of a movement with the actual sensory feedback and as a result downregulates tactile sensitivity when both signals match [8,13]. Here, we investigated how visuomotor predictability influences tactile suppression. To our knowledge, this is the first study that investigates the classic tactile suppression effect in a dynamic interaction task performed in a complex visual environment. In Experiment 1, when participants could not plan their movement trajectory, or had to adjust their movement trajectory unexpectedly, tactile detection thresholds were increased relative to when the movement could be planned. This finding was replicated in Experiment 2, when visual information regarding the position of the hand during movement was removed. These findings are at odds with our hypothesis that tactile suppression should be weaker around the moment of an unexpected perturbation, when increased reliance on somatosensory signals is presumed. Importantly, we found a positive correlation between the detection threshold and the task load that could explain the stronger tactile suppression when visuomotor predictability was low.

Tactile suppression of externally applied stimuli is associated with reduced activation in the secondary somatosensory cortex (SII), the insula and the thalamus [12,51], which are areas involved in somatosensory processing. Reduced activation is also found in the supplementary motor area (SMA) which is related to the generation of efference copy signals which are responsible for the down-regulation of activation in the somatosensory areas [52]. This decrease in activation occurs when predicted sensory feedback from the movement matches the actual sensory feedback [13,53,54]. The decrease in activity in the somatosensory areas and SMA can be modulated by the relevancy of the somatosensory feedback signals for the current task [11]. Moreover, SII and the supramarginal gyrus (SMG) increase their functional connectivity if somatosensory feedback processing becomes more important [11]. It needs to be tested if the Unpredictable Rotation condition compared to the Predictable Rotation and the Stable conditions would result in decreased activation in areas related to somatosensory and efference copy processing and higher functional connectivity between SII and SMG, especially when visual feedback of the hand is missing. Based on our behavioural results it is conceivable that additional cognitive factors, such as task load, might modulate the activation in the tactile suppression network.

In the following, we discuss how the task load might be related to participants' ability to detect a tactile stimulus while moving. We relate these findings to the idea that tactile suppression might occur as a way to free up processing capacities for task-relevant information by downweighting task-irrelevant information [42,44]. We then go on to discuss some of the limitations of using VR for tactile suppression research.

### 9.1. Task load

Typically, we perform more than one task at a time, such as driving and listening to music. Previous research has found that multitasking is associated with increased cognitive demands that lead to a decrease in task performance compared to performing a single task (e.g. [55,56]). It has been suggested that tactile suppression might be one potential mechanism to free up cognitive resources to process novel or more relevant stimuli [42,44]. For example, Gertz *et al*. [57] found no difference in tactile suppression between a dual task, where participants had to detect a vibration on a moving hand that is reaching to a briefly flashed visual target, and a triple task where they additionally had to detect if this visual target flashed once or twice [57]. It is important to note that task performance was overall very high indicating similar task difficulty that might have led to comparable suppression effects.

In both experiments of the current study, the Unpredictable Rotation condition was reported as the most difficult in that it had the highest Global Task Load score as well as the highest proportion of mistrials. This is likely caused by the sudden movement correction participants made in response to the unexpected object rotation, as shown in the movement kinematics, and that interacting with virtual objects of unfamiliar dynamics can increase task load [26]. It might also be caused by the fact that this condition had twice the number of trials as the Predictable Rotation and the Predictable Stable condition, respectively. The higher task load was positively correlated with the tactile detection thresholds where, as the task load increased, tactile sensitivity became worse. This is in line with previous findings showing that the degree of suppression can be modulated by executive capacities [43]. If a task is more difficult, the executive load should be higher and could result in a decreased ability to detect vibrations. Such modulation works on top of the predictive movement-related tactile suppression leading to stronger suppression for movements associated with high than low cognitive demands (e.g. [43,58]). It is possible that the increased suppression with task load is a by-product of the overall number of trials within a condition, and thus it may not reflect the underlying mechanisms of movement-related predictive suppression. Overall, the results from the two experiments suggest that task load is related to our ability to detect tactile stimuli with higher load impeding tactile detection.

Another critical factor is the time of online movement corrections relative to the moment when we probed tactile suppression. Our rationale was that movement correction latencies would arise within 100–150 ms after perturbation onset (e.g. [33,35]), and so by probing tactile suppression around that moment we could capture the period during which a possible dynamic weighting of somatosensory signals from the moving hand could take place. However, our results suggest that the movement corrections have often started *before* the moment we probed suppression. We acknowledge that defining the moment of an online motor correction can depend on various factors (e.g. [35]), but it is likely that part of the modulation of the strength of tactile suppression arises from differences in the timing of our tactile stimulus relative to the motor correction.

### 9.2. Task-irrelevant sensations

In the current experiment, participants performed two tasks: the interception movement and the tactile detection tasks. Given the dynamic nature of the interception task participants might have prioritized this task as their primary task, particularly because the vibration task did not occur unless the participant was moving towards the target, though participants were not made explicitly aware of this. As the interception task becomes harder and requires more processing capacities, either when participants had to change their movement trajectory or when they could not see their hand, they might have further prioritized the interception movement-related information and as a consequence detected the tactile stimulus less frequently. This would still suggest that participants do not prioritize the somatosensory signals from their moving hand, but rather the visual signals about the target position. It is possible that participants may also have some visual memory of their hand position, once the visual cues are removed. However, once the hand starts moving, any visual memory should be eliminated, or substantially reduced. If the vibration, or other tactile information, was informative to the movement task, participants might have detected more vibrations as task difficulty increased, similar to Voudouris & Fiehler [5] where task-relevant vibrations were enhanced and task-irrelevant ones were suppressed [5] or similar to Manzone, Tremblay and Chua [59] who found that the expectation of intercepting a real world object led to lower detection thresholds [59].

### 9.3. The use of virtual reality

This experiment extends previous work showing that off the shelf VR equipment can be used to investigate tactile suppression using a psychophysical task [24]. It also extends previous research on tactile suppression to more naturalistic settings by using large-scale movements in a dynamic interaction task performed in a complex visual environment. However, it is not without its limitations. The VR controller in this experiment could not produce a vibration below 0.0009 (arb.units) which for some participants was not low enough to produce vibrations below their detection threshold. Further, the vibration is applied to the complete palm, and therefore these might feel different depending on how strongly one holds the controller (stronger grip, better and larger contact area, better detection). One might speculate that participants would hold the controller with greater force when having to navigate a perturbation, and with even more force when this perturbation would be unexpected, and thereby, this potential difference might explain the obtained results. This is rather unlikely, not only because the amount of produced force does not seem to influence tactile perception [17], but also because a stronger grip in the unpredictable conditions would increase the contact area between the controller and the moving hand, thereby leading to *better* and not *worse* (as we found) tactile detection.

As all stimuli in this experiment were visual, except for the applied vibration, tactile signals were probably not used to determine a successful hit of the target object. This is in contrast to real world interactions where haptic feedback is present when making contact with an object. During a motor correction to an unpredictable visual perturbation, we likely attend more to somatosensory cues when no visual feedback of the hands is provided. These cues are not necessarily tactile cues but could also be proprioceptive. However, tactile cues are still important for movement control, as the absence of the tactile sense can lead to abnormal reaching and grasping behaviour [60]. Moreover, tactile sensitivity during movement is modulated even at moments when tactile signals from the environment are absent [23]. While studies have found differences in tactile suppression when comparing between conditions with and without object contact [61], it is possible that having a predictable physical consequence to the end of our movement would lead to similar levels of suppression in all of our conditions [14].

Nevertheless, we believe the lack of a physical consequence to the movements in these experiments would likely not affect the interpretation of our results. If the inability to receive haptic feedback when physically interacting with the stimuli affected tactile detection, we would expect it to similarly affect all movement conditions, not just the rotation conditions.

## 10. Conclusion

For the first time, we demonstrate the classic tactile suppression effect in a dynamic interaction task performed in a complex visual environment. Contrary to previous findings [22], we observed stronger tactile suppression when visuomotor predictability was decreased. Importantly, low predictability was associated with high task load that correlated with the strength of tactile suppression. Our results show that task load can influence tactile suppression, possibly by downweighting task-irrelevant somatosensory feedback signals to allow for successful task performance when visuomotor demands are high. Future studies on movement-induced tactile suppression need to consider potential differences in task load when interpreting their results.

## Data Availability

The data collected for this work as well as the related analysis routines are publicly available at: https://doi.org/10.17605/OSF.IO/G5CPQ [62].
